# Embracing Digital Health Innovations to Enhance Universal Health Coverage Through SMART Health Governance

**DOI:** 10.34172/hpp.45788

**Published:** 2026-08-21

**Authors:** Susan S. Francis, Vijay Kumar Chattu

**Affiliations:** ^1^Center for Evidence-based Research, Global Health Research and Innovations Canada Inc. (GHRIC), Toronto, ON, Canada; ^2^United Nations University Institute on Comparative Regional Integration Studies (UNU-CRIS), Potterierei 72, 8000 Bruges, Belgium; ^3^School of Public Health, University of Alberta, Edmonton, AB T6G 1C9, Canada; ^4^Central Research Facility (CRF), Dr. D.Y Patil Medical College, Hospital and Research Centre, Dr. D.Y Patil Vidyapeeth, Pune- 411018, India

**Keywords:** Digital health, Public health, Equity, Universal health coverage, Expenditure, Governance

## Abstract

Universal Health Coverage (UHC) is one of the core objectives of global health and development programs worldwide, but progress remains uneven and fragile, particularly in low- and middle-income countries (LMICs). This perspective paper examines the role of digital health innovations (DHIs) in improving UHC and argues that their impact is shaped by the quality of governance under which they are designed and implemented. DHIs have increasingly been promoted as mechanisms to improve access, efficiency, transparency, and continuity of care. Yet across several LMICs, DHIs are often implemented in a fragmented manner, limiting their contribution to equitable, sustainable progress toward UHC. The paper highlights how governance attributes such as accountability, transparency, responsiveness, and ethical oversight influence digital health outcomes. It also discusses how smart health governance, characterized by simple, moral, accountable, responsive, and transparent institutional arrangements, is essential to align digital health investments with national health priorities and equity objectives. The paper argues that integrating digital health innovation with governance reform is critical for strengthening health system performance and achieving sustainable progress toward UHC for all.

## Introduction

 Universal Health Coverage (UHC) is widely recognized as a central objective of global health and development policy. Despite sustained international commitment and funding, the progress toward UHC remains uneven across many regions. Globally, about 4.5 billion people continue to lack access to essential health services as of 2021, and improvements in service coverage have slowed in recent years. Millions of households are experiencing catastrophic and impoverishing health expenditures.^1^ These global patterns indicate that the challenge of achieving UHC lies not in the absence of policy intent, but in persistent weaknesses in health system design, financing, and governance. UHC is explicitly articulated under Sustainable Development Goal (SDG) target 3.8, which calls for access to quality essential health-care services and financial risk protection for all.^2^ SDG 3.b emphasizes the need to develop medicines to address persistent treatment gaps.^3^ Out-of-pocket (OOP) expenditure is an important indicator of weak financial protection, particularly in low- and middle-income countries (LMICs). In many LMICs, OOP payments amount to a high share of total health expenditure, exposing households to catastrophic health spending and medical impoverishment, and affecting poor households, informal workers, rural populations, and women.^1^ Persistently high OOP expenditure reflects structural deficiencies within health systems, including inadequate risk pooling and fragmented financing in primary health care. Given this context, Chattu et al. highlighted that Health as a human right obligates states to ensure access to timely, acceptable, and affordable health care, including access to essential medicines.^4^

 Coverage gaps are particularly evident in the management of noncommunicable diseases, mental health services, and rehabilitative care. These challenges are not entirely technical but closely linked to governance failures, including structural deficiencies in stewardship, regulation, incentive alignment, and accountability. In response to these gaps, digital health innovations (DHIs) have increasingly been advanced as mechanisms to improve access, efficiency, and coordination within health systems. When digital tools are introduced without alignment with financing, service delivery, and regulatory frameworks, they may reinforce existing inequities rather than addressing underlying system constraints.^5^ Governance, therefore, plays a decisive role in shaping whether digital health investments translate into meaningful progress toward UHC. Weak governance can result in poorly integrated digital ecosystems, poor interoperability, inadequate data protection, and exclusion of populations with limited digital access. Effective governance aligns digital health initiatives with national health priorities, strengthening accountability and promoting equitable access. This highlights the need for smart health governance that is simple, ethical, accountable, responsive, and transparent to guide digital health adoption and scale. Therefore, given this background, this paper aimsto examine how digital health innovations, when embedded within smart health governance frameworks, can address persistent system gaps and advance progress toward UHC, particularly in LMICs. The paper examines the relationship between DHIs, governance reform, and UHC, particularly in LMICs, and the discussion draws on published literature, global health reports, policy frameworks, and illustrative examples relevant to digital health governance, equity, and health systems strengthening in LMICs.

## What is UHC, and How Does it Matter?

 UHC refers to the ability of all people to access health services from health promotion and disease prevention to treatment, rehabilitation, and palliation without financial hardship. It is not limited to expanding service availability or insurance enrolment; it emphasizes quality, equity, and financial protection and serves as a measure of overall health system performance and social solidarity.^1^ The significance of UHC extends beyond SDG 3, and overlaps with broader development priorities such as poverty reduction (SDG 1), gender equality (SDG 5), reduced inequalities (SDG 10), and inclusive economic growth (SDG 8). Progress toward UHC is therefore foundational to achieving the wider 2030 Agenda for Sustainable Development. Limited advancement curtails progress towards UHC and perpetuates existing social and economic inequities.^2^ Health systems that achieve UHC are associated with lower mortality, improved population health outcomes, and reduced socioeconomic disparities. Financial risk protection is particularly critical, as exposure to high out-of-pocket payments can push households into poverty and deter timely care-seeking.^1^ SDG 3.8 specifies “access to safe, effective, quality and affordable essential medicines and vaccines for all” as a central component of UHC.^6^ Other contributions of UHC include broader economic and social goals, such as improving workforce productivity, reducing long-term public expenditure on preventable complications, and strengthening trust in public institutions. Besides, it was highlighted that the countries and states that progress towards achieving UHC will also make progress towards other health-related SDGs.^7^

## Bottlenecks in Achieving UHC and Where Do LMICs Stand?

 Achieving UHC is limited by several interrelated bottlenecks that extend beyond resource limitations. Fragmented health financing systems feature multiple pools and weak risk-sharing mechanisms, threatening financial protection and equity. Limited strategic purchasing capacity and weak provider payment mechanisms reduce efficiency and weaken incentives for quality improvement.^6^ Health workforce constraints, such as persistent shortages, inequitable distribution, and inadequate skills, limit service availability and quality, especially in primary care. Weak referral systems and an underdeveloped health information infrastructure also contribute to fragmented service delivery and poor continuity of care. Governance challenges, including weak stewardship, insufficient regulatory oversight, and limited accountability across public and private settings, magnify these technical constraints.

 LMICs have made uneven progress toward health-related SDGs, particularly under SDG target 3.8. While some LMICs have expanded service coverage and shown improvements in specific disease control programs, many have yet to meet health-related SDG targets by 2030.^2^ Progress has been especially limited in areas such as primary health care, noncommunicable disease management, and mental health services. Several structural constraints, such as limited fiscal space, high reliance on out-of-pocket payments, and fragmented health financing arrangements, limit LMICs’ ability to achieve equitable coverage. In this context, it was highlighted that, globally, access to medicines is increasingly a multidimensional and multisectoral public health issue^8^, and the African Health Strategy 2016-30 also emphasized that UHC must be strengthened by adequate supply of commodities such as essential medicines, vaccines, contraceptives, drugs, and other supplies.^9^ Health workforce shortages and maldistribution also diminish service availability, particularly in rural and underserved areas.

## Emerging Role of Digital Health Innovations

 DHIs are increasingly recognized as enablers of health system strengthening towards achieving UHC, particularly in LMICs. The World Health Organization Global Strategy on Digital Health 2020–2027 provides a normative framework for the adoption and scale-up of digital technologies to support equitable, people-centered health systems. This strategy emphasizes country ownership, interoperability, equity, and the responsible use of health data, positioning digital health as a means to improve system performance rather than an end in itself.^10^

 When effectively integrated into health systems, DHIs can improve access through telemedicine, remote diagnostics, and digital appointment systems, particularly for rural and underserved populations. They can also improve affordability and efficiency by reducing administrative costs, streamlining claims processing, and improving supply chain management. These platforms also contribute to faster service delivery and greater transparency, enabling real-time data reporting, improved referral coordination, and better tracking of public health expenditures and outcomes.^10^ Digital transformation also introduces significant risks, including data privacy, cybersecurity, algorithmic bias, and exclusion of populations with limited digital access. These can dilute trust and exacerbate inequities if not adequately addressed. Regulatory frameworks, therefore, play a critical role. In India, the Digital Personal Data Protection (DPDP) Act establishes legal safeguards for personal data, including health information, while enabling data use under defined conditions.^11^ Internationally, frameworks such as the European Union’s General Data Protection Regulation (GDPR) illustrate approaches to balancing innovation with individual rights and accountability.^12^ These examples highlight that the benefits of digital health for UHC are contingent on robust governance and data protection mechanisms.

## SMART Health Governance as The Key

 Simple, Moral, Accountable, Responsive, Transparent (SMART) health governance is the integration of digital technologies, AI, and data-driven methods into public administration to enhance efficiency, transparency, and citizen engagement. It fosters collaboration between stakeholders to solve urban challenges, reduce bureaucracy, and provide better public services.^13^ Janssen and Estevez defined SMART health governance as governance arrangements that integrate digital technologies, institutional accountability, ethical oversight, and data-driven decision-making to improve health system performance, transparency, and equitable service delivery.^14^

###  Key Aspects of SMART Governance

 The SMART governance (Figure 1) framework can be operationalized within health systems through measurable governance functions and implementation mechanisms.

**Figure 1:**
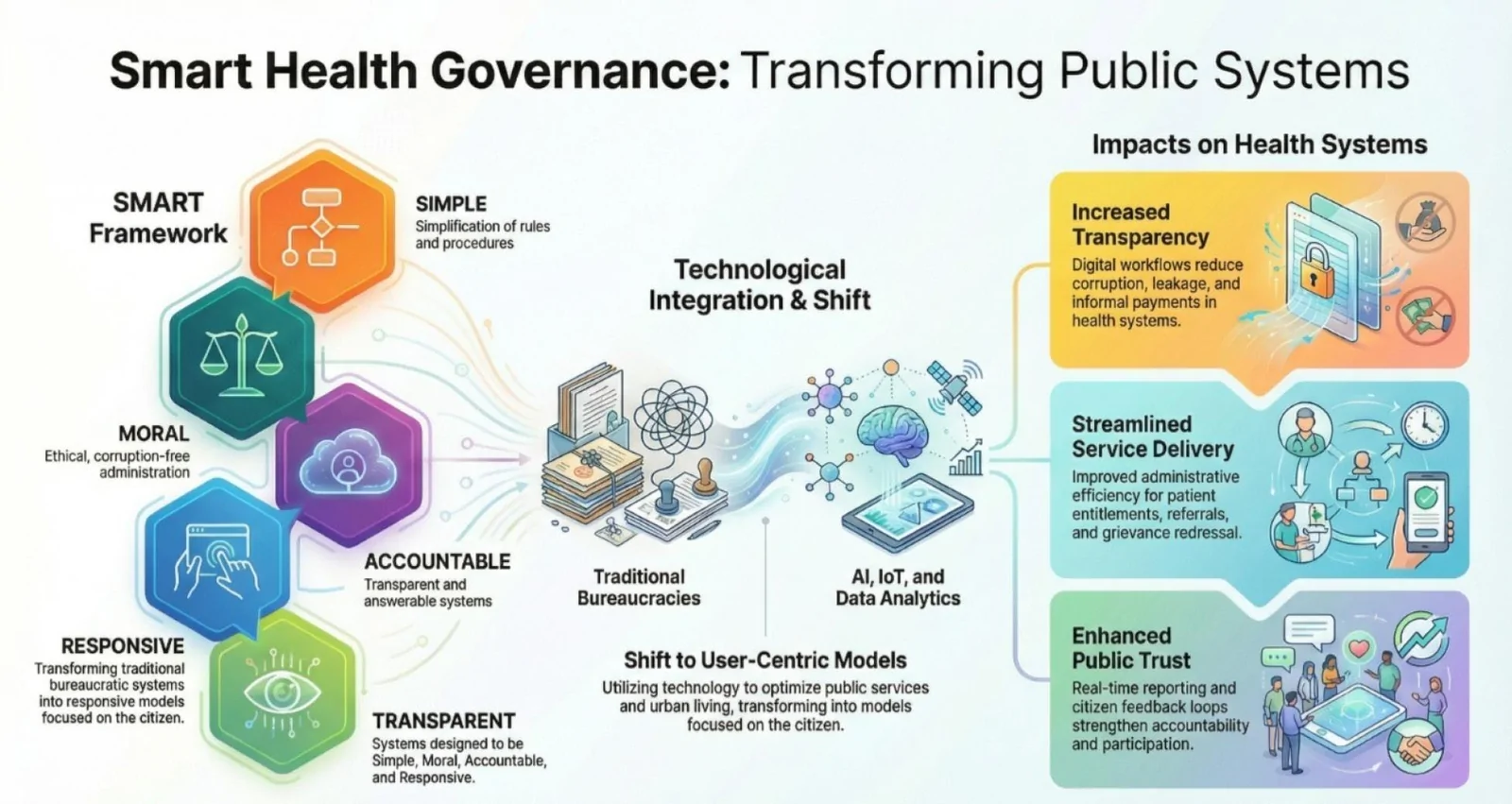
Smart Governance Framework for Health Systems Transformation

####  Applications

 Several LMICs have initiated governance reforms aligned with SMART health governance principles. India’s Ayushman Bharat Digital Mission (ABDM) aims to strengthen interoperability and continuity of care through an integrated digital health infrastructure linking providers, patients, and health records.^15^ Rwanda has similarly expanded telemedicine and digital health initiatives to improve access to services and coordination in underserved settings.^16^ These experiences suggest that digital transformation is more effective when supported by institutional coordination, regulatory stewardship, and accountability mechanisms rather than isolated technological implementation. Other broader examples include Smart city planning ^17^, digital payment systems (G2P)^18^, and online service delivery portals, e.g., K-SMART in Kerala.^19^

 These approaches transform traditional, bureaucratic systems into responsive, user-centric models, often serving as a pillar of smart cities.

 Source: Image generated by the authors using ChatGPT from the prompt “Conceptual figure generated with support from Generative AI (ChatGPT, OpenAI) using the prompt below.” Create a structured academic infographic titled “Smart Health Governance: Transforming Public Systems”.

 The figure should illustrate:

- A SMART governance framework comprising: Simple, Moral, Accountable, Responsive, and Transparent principles.
- A transformation pathway from Traditional Bureaucratic Models to digitally enabled systems incorporating AI, IoT, and Data Analytics, labeled “Technological Integration & Shift.”
- A transition toward User-Centric Service Delivery Models.
- Key Health System Impacts: Increased Transparency, Streamlined Service Delivery, and Enhanced Public Trust.

The visual should be clear, logically organized, and appropriate for inclusion as a conceptual framework.

###  Key Benefits of Smart Governance in Health Systems

 Smart governance frameworks offer several interrelated benefits for health systems, particularly in LMICs, where administrative inefficiencies and weak accountability mechanisms have historically constrained service delivery.

1. One of the most significant benefits is increased transparency through digital workflows, real-time reporting, and auditable data trails. By reducing reliance on discretionary decision-making and fragmented manual processes, smart governance may help reduce leakage, informal payments, and inefficiencies that continue to affect equitable access in many LMIC health systems.^20^
2. Smart governance also contributes to improved service delivery by streamlining administrative procedures, reducing delays, and enhancing coordination across different levels of government and service providers. Digitally enabled governance platforms improve administrative efficiency by improving entitlements, referrals, and grievance redressal in resource-constrained public health systems.^21^
3. Another critical benefit is enhanced citizen participation and trust. Smart governance models incorporate citizen feedback, service tracking, and participatory monitoring, enhancing transparency, responsiveness, accountability, and improving public trust. Smart governance supports sustainable development by improving the efficiency and effectiveness of public spending through better data integration, performance monitoring, and strategic resource allocation.^22^

###  Applications of Smart Governance in LMIC Contexts

 Smart governance in many LMICs is increasingly being put into practice through digital public infrastructure that connects health, sanitation, transport, and social services, enabling urban governments to plan and deliver services more coherently and with greater information.^22^ Digital government-to-person payment systems have helped governments deliver health and social benefits directly to individuals, reducing delays, administrative costs, and leakage while strengthening financial protection for populations that are often hardest to reach.^23^ Several LMICs have initiated governance reforms aligned with SMART health governance principles. India’s Ayushman Bharat Digital Mission (ABDM) aims to strengthen interoperability and continuity of care through an integrated digital health infrastructure linking providers, patients, and health records. Rwanda has similarly expanded telemedicine and digital health initiatives to improve access to services and coordination in underserved settings. These examples illustrate how digital transformation may support health system strengthening when accompanied by institutional coordination, accountability mechanisms, and regulatory stewardship. Online service delivery portals, including subnational platforms in India and similar initiatives in parts of Africa, Eastern Europe, and Latin America, have made it easier for people to access public services, track applications, and raise grievances, improving transparency and accountability in everyday interactions.^24^

###  Smart Governance and Digital Health Innovations as a Key Strategy for UHC

 Smart governance combined with DHIs may contribute to progress toward UHC by improving system efficiency, accountability, and equity. Digital health interventions such as telemedicine, interoperable health records, and digital claims systems have been associated with improvements in service coordination, continuity of care, and administrative efficiency in several LMIC settings.^3^ These benefits, however, depend on governance quality, interoperability, institutional capacity, and equitable implementation.

 Source: Conceptual figure generated with support from Generative AI (ChatGPT, OpenAI) using the prompt below.

 Create a professional academic conceptual framework diagram titled “Role of Digital Health Innovations in Strengthening Universal Health Coverage (UHC) in LMICs.”

 Structure it vertically with four sections:

1. Key LMIC challenges (high out-of-pocket costs, access gaps, workforce shortages, fragmented governance);
2. Digital health interventions (telemedicine, digital records, e-claims, referral systems, supply chain digitization, data platforms);
3. System outcomes (improved access, financial protection, efficiency, equity, transparency);
4. Impact pathway showing: Digital Health + Smart Governance → Stronger Health Systems → Accelerated UHC.

Use a clean, journal-quality layout with minimal icons, muted blue/grey tones, white background, clear arrows, and no decorative or cartoon elements.

 When embedded within strong governance frameworks, DHIs may support improvements in access to primary care, continuity of care, disease surveillance, and referral coordination (Figure 2). Digital tools supporting referrals, clinical decision-making, and population-level monitoring have been associated with improved service delivery and strengthened health system responsiveness in several settings.^1^ These benefits depend on effective public stewardship. Governments must ensure that digital health initiatives are guided by clear regulatory frameworks, accountability mechanisms, and a focus on public value to avoid fragmentation and inequity. Equity-centered governance is essential to ensure that digital transformation includes vulnerable and historically marginalized groups, including indigenous populations, women, children, persons with disabilities, and LGBTQ + communities, and translates into improved access, financial protection, and health outcomes.

**Figure 2:**
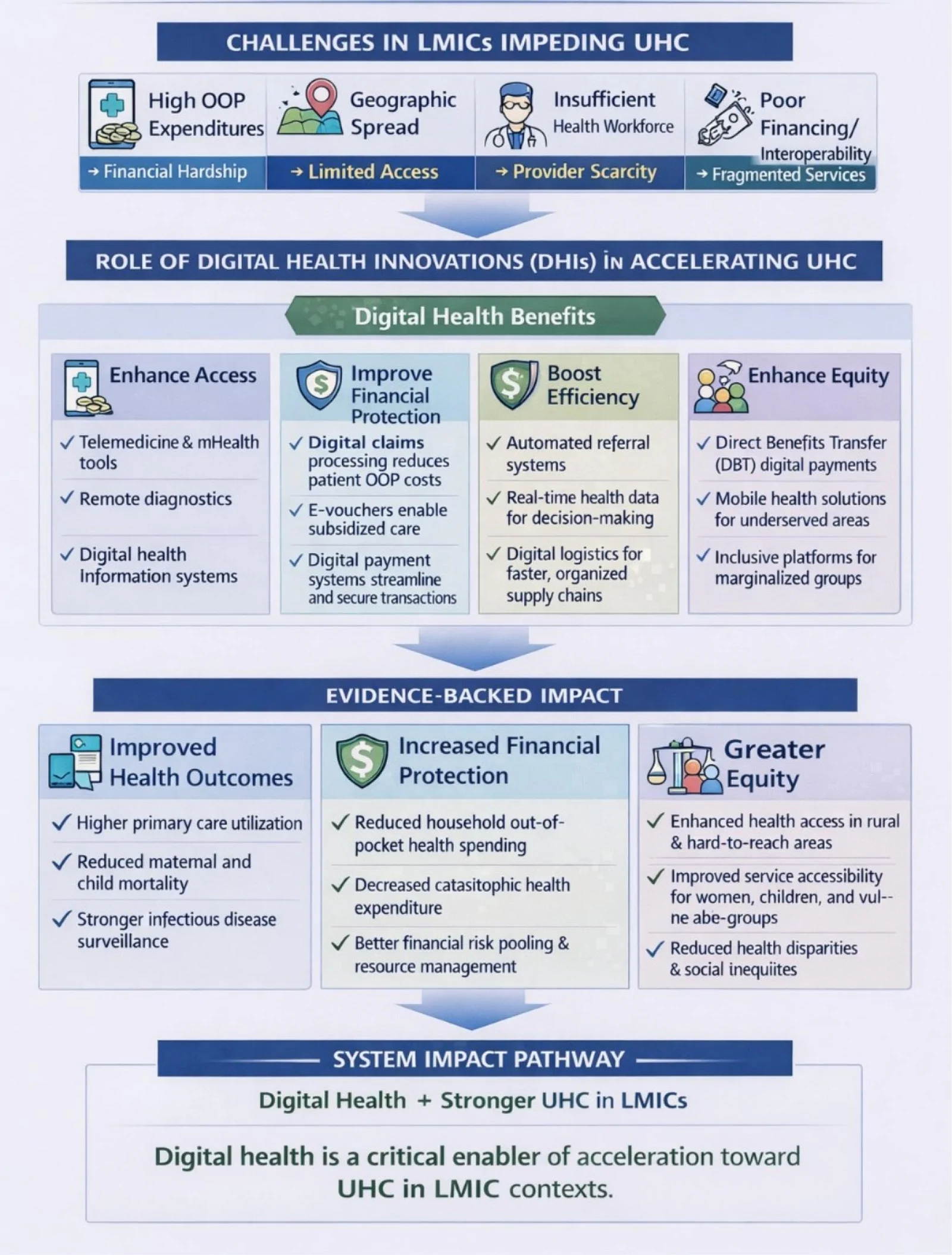
Role of Digital Health Innovations for enhancing Universal Health Coverage

 Governments should also invest in digital literacy initiatives, subsidized internet connectivity, multilingual platforms, and low-bandwidth or offline service alternatives to reduce digital exclusion. Monitoring indicators such as rural digital service utilization, gender differences in digital access, and accessibility for persons with disabilities should also be incorporated into digital health governance frameworks.

## Limitations and Implementation Risks

 Despite the potential benefits of digital health innovations and SMART governance frameworks, several implementation challenges may limit their effectiveness and sustainability, particularly in LMIC settings. Digital transformation initiatives often require substantial investments in infrastructure, workforce training, interoperability systems, cybersecurity protections, and long-term maintenance.^23^ Weak interoperability, vendor lock-in, limited institutional capacity, and digital exclusion may reduce effectiveness and sustainability if not addressed through phased implementation strategies and regulatory oversight. Some policy recommendations for implementation in LMICs are listed below (Table 1).

**Table 1:** Policy Recommendations for governments of Low-Middle-Income Countries

**Priority Area**	**Recommended Action**
Digital Health Infrastructure	Develop interoperable national digital health systems that integrate health records, referral pathways, and claims management platforms
Data Governance and Regulation	Strengthen legal and regulatory frameworks related to data protection, cybersecurity, interoperability standards, and ethical use of digital health technologies
Equity and Digital Inclusion	Invest in digital literacy programs, subsidized connectivity, multilingual interfaces, and low-bandwidth or offline service alternatives for underserved populations
Strategic Purchasing and Financing	Align digital health investments with broader UHC financing reforms and strategic purchasing mechanisms to improve efficiency and accountability
Accountability and Transparency	Establish measurable governance indicators, grievance redressal systems, and public reporting mechanisms to improve institutional accountability
Workforce Capacity	Strengthen workforce training in digital governance, health informatics, and implementation management across health systems

Source: Prepared by the authors.

## Conclusion

 Persistent gaps in UHC are driven not only by financial constraints but also by weaknesses in health system design, governance, and accountability. DHIs can improve access, efficiency, and transparency, yet their contribution to UHC remains limited when implemented in fragmented ways and without effective stewardship. Aligning digital transformation with governance reform is therefore critical for achieving equitable and sustainable progress toward UHC, particularly in LMICs.Governments should integrate digital health initiatives into comprehensive health systems and governance reforms, ensuring interoperability, data protection, and accountability, while aligning investments with UHC priorities. Health policies should adopt an explicit equity lens, prioritizing vulnerable and marginalized populations to ensure that digital health and governance reforms reduce, rather than reinforce, existing disparities.

## Competing Interests

 Vijay Kumar Chattu is an editorial board member of Health Promotion Perspectives. All the other authors declare no competing interests in this work.

## Data Availability Statement

 Data is available from the corresponding author upon request.

## Ethical Approval

 Not applicable.
